# The Influence of Stride Selection on Gait Parameters Collected with Inertial Sensors

**DOI:** 10.3390/s23042002

**Published:** 2023-02-10

**Authors:** Carmen J. Ensink, Katrijn Smulders, Jolien J. E. Warnar, Noël L. W. Keijsers

**Affiliations:** 1 Department of Research, Sint Maartenskliniek, 6500 GM Nijmegen, The Netherlands; 2 Department of Sensorimotor Neuroscience, Donders Institute for Brain, Cognition and Behaviour, Radboud University, 6500 HB Nijmegen, The Netherlands; 3 Department of Rehabilitation, Donders Institute for Brain, Cognition and Behaviour, Radboud University Medical Center, 6500 HB Nijmegen, The Netherlands

**Keywords:** gait analysis, inertial measurement units, steady-state gait, turning, stride selection

## Abstract

Different methods exist to select strides that represent preferred, steady-state gait. The aim of this study was to identify the effect of different stride-selection methods on spatiotemporal gait parameters to analyze steady-state gait. A total of 191 patients with hip or knee osteoarthritis (aged 38–85) wearing inertial sensors walked back and forth over 10 m for two minutes. After the removal of strides in turns, five stride-selection methods were compared: (*ALL*) include all strides, others removed (*REFERENCE*) two strides around turns, (*ONE*) one stride around turns, (*LENGTH*) strides <63% of median stride length, and (*SPEED*) strides that fall outside the 95% confidence interval of gait speed over the strides included in *REFERENCE*. Means and SDs of gait parameters were compared for each trial against the most conservative definition (*REFERENCE*). *ONE* and *SPEED* definitions resulted in similar means and SDs compared to *REFERENCE*, while *ALL* and *LENGTH* definitions resulted in substantially higher SDs of all gait parameters. An in-depth analysis of individual strides showed that the first two strides after and last two strides before a turn were significantly different from steady-state walking. Therefore, it is suggested to exclude the first two strides around turns to assess steady-state gait.

## 1. Introduction

Gait is one of the most fundamental activities of daily life. Unsurprisingly, gait impairments negatively impact independent living and the quality of life of individuals [1]. Gait capacity is commonly described by the means and variability of spatiotemporal gait parameters during steady-state walking. While the mean gait speed is widely accepted as an indicator of overall gait capacity, the variability of spatiotemporal gait parameters is associated with dynamic balance [2,3]. However, to adequately quantify the measures of variability, a substantially higher number of steps needs to be analyzed than is typically recorded in overground gait labs using optical motion analysis [4]. Inertial measurement units (IMUs) can be used to record a multitude of steps per trial, with the additional advantage that they can be used outside the lab, in more ecologically valid settings and in real life [3,5]. However, as testing space in the clinic can be limited, gait assessments typically include back-and-forth walking, including turns. The acceleration and deceleration phases associated with these turns can substantially influence the mean and variability of spatiotemporal gait parameters [4]. Therefore, to characterize straight-ahead gait, only the strides in the steady-state portion of gait should be included for analysis, thus discarding the strides made in turns and during acceleration and deceleration phases. Although the validity of gait event detection and estimation of spatiotemporal gait parameters using IMUs has received ample attention [6,7,8,9], these studies did not evaluate how choices regarding the exclusion of strides in turns and periods of acceleration and deceleration affect spatiotemporal gait parameters during steady-state gait.

Stride-selection methods presented in the literature are based on two main methods. Either a fixed number of strides are excluded around turns or after starting [10], or strides are identified based on a certain relative threshold, e.g., minimum stride length [11]. As yet, it is unclear to what extent different methods to select strides affect the calculated means and variance of spatiotemporal gait parameters. Therefore, the aim of this study was to compare methods to select strides representative of steady-state, straight-ahead gait. Our first research question was: what is the effect of stride-selection methods on the means and variability of spatiotemporal gait parameters in tests including turns? The second research question was: how much do strides preceding and directly following the turns deviate from the steady-state portion of the walking trajectory? We analyzed these strides in more depth to understand the effect of acceleration and deceleration phases on the observed difference between selection methods. For this study, people with osteoarthritis (OA) of the lower limb joints or joint replacement after OA were included. OA of the lower limb joints is a well-known cause of impaired gait capacity [12,13]. People with OA, for example, walk with a lower gait speed compared to their healthy peers [13], but without the more severe impairments, such as freezing of gait or drop foot related to neurological diseases.

## 2. Materials and Methods

### 2.1. Subjects

Participants were recruited from the outpatient clinic of the orthopedic department of the Sint Maartenskliniek between October 2020 and October 2021. They were invited to participate if they had visited the clinic for end-stage knee, hip or ankle OA confirmed by an orthopedic surgeon, or after total knee or hip arthroplasty (TKA or THA) due to OA. Participants had to be at least 18 years old. People were excluded if they had gait or balance problems caused by anything other than OA. Informed consent was obtained from each participant prior to testing. A total of 191 people participated in this study, and eight people participated twice; before and after joint replacement surgery. This resulted in a total of 199 measurements that were analyzed.

### 2.2. Gait Assessment

**Figure 1:**
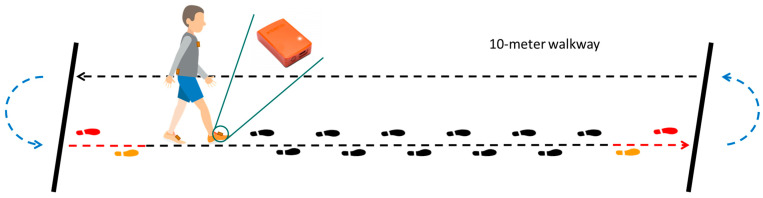
Set-up for 2 min walking test. Participants were instructed to turn after the 10 m marks (thick, black lines), but no specific instructions on how to turn were given (e.g., pivot turn or taking multiple steps). Orange and red indicate the first and second foot strike after a turn (blue) and the last and second to last foot strike before the turn start, respectively.

Participants were equipped with four IMUs (Xsens Awinda, Enschede, The Netherlands) placed on both feet (dorsum side of the foot), the upper part of the sternum, and the lumbar level (L4/L5) of the trunk. Subsequently, participants walked back and forth over 10 m in a broad hallway in the clinic, performing 180° turns after each 10 m stretch (Figure 1). They were instructed to walk for two minutes at a self-selected, comfortable pace and to turn beyond the 10 m mark (line). No specific instructions were given on how to turn (e.g., pivot turn or taking multiple steps). Measurements were captured with MTManager software suite (version 2019.2) at 100 Hz. The planning, conduct and reporting of this study was in line with the Declaration of Helsinki. The study protocol was approved by the institutional review board.

### 2.3. Stride Identification

**Figure 2:**
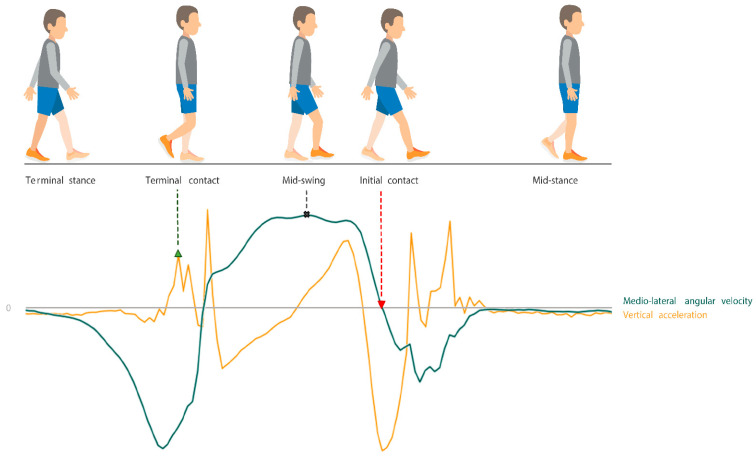
Stride identification was performed based on the filtered mediolateral angular velocity (green graph) and vertical free-acceleration (orange graph) signal features of the foot sensors. Terminal contact was determined at the local peak in vertical acceleration (green triangle pointing up), mid-swing at the peak in medio-lateral angular velocity (cross), and initial contact at zero-crossing of the medio-lateral angular velocity (red triangle pointing down).

**Figure 3:**
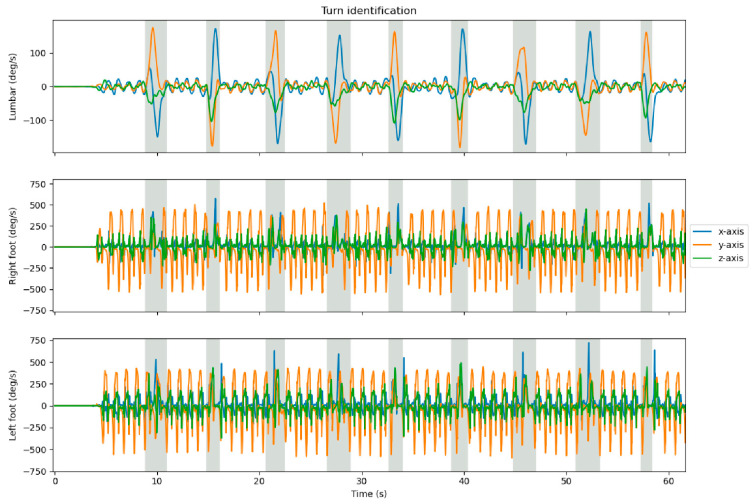
Turns (grey areas) were identified at the local maxima of the absolute angular velocity (rotated to the earth frame) around the vertical axis (green z-axis) of the lumbar sensor (top graph) [11]. The start of a turn was defined as the last instant that the absolute angular velocity around the vertical axis was <5°/s. The finish of each turn was defined as the last instant at which the absolute angular velocity was >5°/s [11].

The identification of initial contact [7] and terminal contact [14], as well as calculating the resulting stride-by-stride spatiotemporal parameters [9] and detection of turns [15] was performed using previously validated algorithms [7,9,14,15]. First, the raw data of the IMUs attached to the feet were filtered by a second-order low-pass Butterworth filter (15 Hz cut-off frequency for angular velocity, and 17 Hz cut-off frequency for acceleration) [7]. Next, mid-swing was identified at the local maximum (clockwise direction) of the filtered angular velocity around the mediolateral axis (flexion–extension movement), directly followed by the zero-crossing (negative slope) corresponding to initial contact [7] (Figure 2). Terminal contact was identified at the peak in the filtered, vertical free acceleration of the IMUs on the feet before the identified mid-swing [14]. In case multiple peaks were identified, the peak with the smallest angular velocity was considered the true terminal contact. To identify turns, the angular velocity of the IMU on the lumbar level was rotated to the earth frame, after which the maxima were detected around the absolute vertical axis [15] (Figure 3). The start of a turn was defined as the last instant that the absolute angular velocity around the vertical axis was <5°/s. The finish of each turn was defined as the last instant at which the absolute angular velocity was >5°/s [15]. Linear velocity was calculated by integrating the free acceleration of the IMUs on the feet. To eliminate drift in the linear velocity and resulting position estimation, zero velocity updates were performed during mid-stance [16,17,18]. Position estimation was performed by integrating the zero-velocity updated linear velocity. All resulting spatiotemporal parameters were calculated on a stride-by-stride basis. The stride time was calculated as the time between two consecutive initial contacts. The gait speed was calculated as the average velocity between two consecutive initial contacts. The stride length was calculated as the absolute difference in position between a terminal contact and the following initial contact.

Five definitions to include strides representative for steady-state gait were compared. Before applying any definition, all strides made within turns were removed. Definitions are the following:

1. *ALL*: Include all strides;
2. *REFERENCE*: Remove first 2 strides after, and last 2 strides before turn [10];
3. *ONE*: Remove first stride after, and last stride before turn;
4. *LENGTH*: Filter out strides <63% of median stride length [11];
5. *SPEED*: Calculate mean and 95% confidence interval (95% CI) of gait speed over the strides included in *REFERENCE*, then include all strides within this 95% CI.

### 2.4. Statistical Analysis

For each definition, the means and standard deviation (SD) of gait speed, stride length and stride time over strides were calculated for each trial using Python’s numpy (v1.22.0) package. Definitions were compared against the most conservative definition, *REFERENCE*, using mean differences and their 99% confidence interval (99% CI). For the in-depth analysis, the first four strides after and before a turn were compared with the middle section of the walking trajectory using mean differences and their 99% CI. The middle section of the walking trajectory consists of the fifth stride after the turn, up to and including the fifth stride before the next turn. To explore if age and gait speed differences between participants affected the acceleration and deceleration phases, the research sample was split into tertiles based on age (youngest 33%, middle 33% and oldest 33%) and gait speed achieved in the middle section of the walking trajectory speed (fastest 33%, middle 33% and slowest 33%) (Appendix B). Statistical analysis was performed by shared control mean difference statistical tests (ordered groups ANOVA) of Python’s dabest (v0.3.1) package [19].

## 3. Results

### 3.1. Subject Characteristics

Participants were aged between 38 and 85 years (mean ± SD: 63.1 ± 9.0), and 110 were female and 81 male. In total, 93 measurements were performed in end-stage OA, and 106 were after joint-replacement surgery. See Table 1 for all participant characteristics.

### 3.2. Spatiotemporal Gait Parameters

#### 3.2.1. Comparison between Selection Definitions

**Figure 4:**
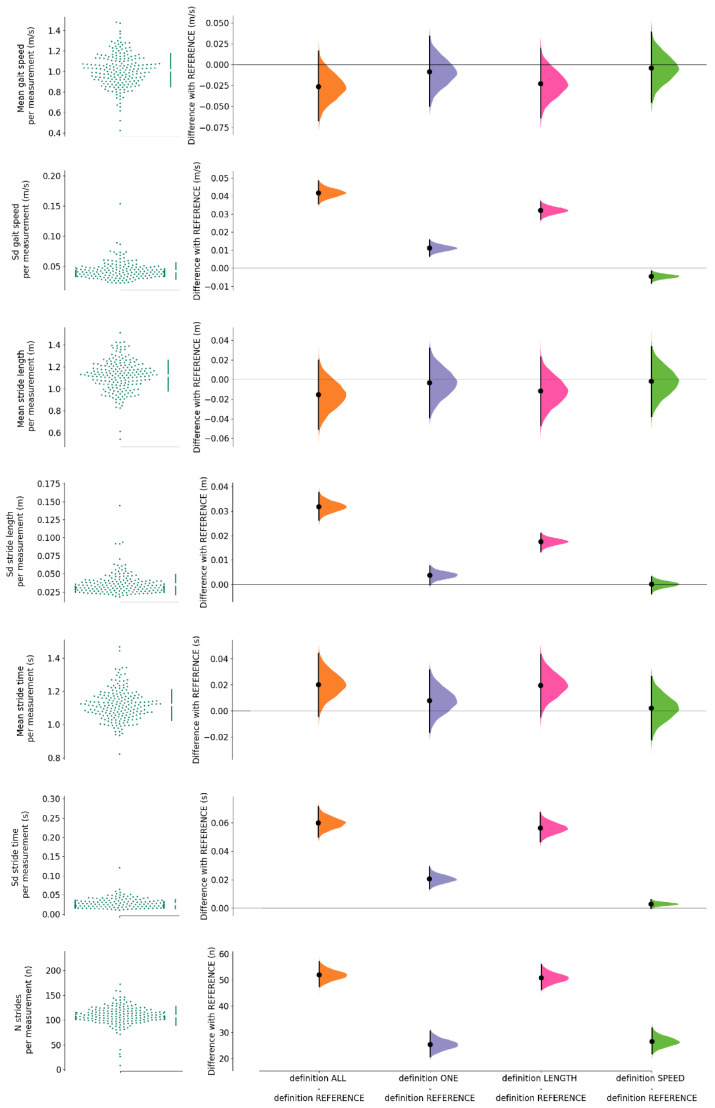
Scatterplot of means and SD of gait speed, stride length and stride time for definition *REFERENCE*, and the mean difference plots for each definition with *REFERENCE*, including the average number ± SD of selected strides per trial for each definition (N).

The average number of selected strides per trial ranged from 108 (*REFERENCE*) to 160 (*ALL*), while the average amount of turns per trial was 10 (range 1 to 19). Means and SDs of gait speed, stride length and stride time for definition *REFERENCE*, and the mean differences and associated 99% CI of each definition with *REFERENCE* are shown in Figure 4. Mean gait speed did not differ from *REFERENCE* for any definition. The SDs of gait speed of all definitions were different compared to *REFERENCE*, ranging from −0.00 (99% CI: −0.01, −0.00) m/s for *SPEED* to 0.04 (99% CI: 0.04, 0.05) m/s for *ALL*. Stride length did not differ from *REFERENCE* for any definition. The SD of stride length differed from *REFERENCE* for all definitions except *SPEED*, ranging from 0.00 (99% CI: 0.00, 0.01) m for *ONE* to 0.03 (99% CI: 0.03, 0.04) m for *ALL*. Definitions *ALL* and *LENGTH* resulted in a significantly higher stride time of 0.02 (99% CI: 0.00, 0.04) s than *REFERENCE*. The stride time derived from *ONE* and *SPEED* did not significantly differ from *REFERENCE*. The SDs of stride time of all definitions were different compared to *REFERENCE*, ranging from 0.00 (99% CI: 0.00, 0.01) s for *SPEED* to 0.06 (99% CI: 0.05, 0.07) s for *ALL*. Table A1 of Appendix A includes all means and SDs of gait speed, stride length, stride time and the number of strides included per definition, as well as their differences with *REFERENCE*.

#### 3.2.2. In-Depth Analysis of Strides around Turns vs. Middle Section

**Figure 5:**
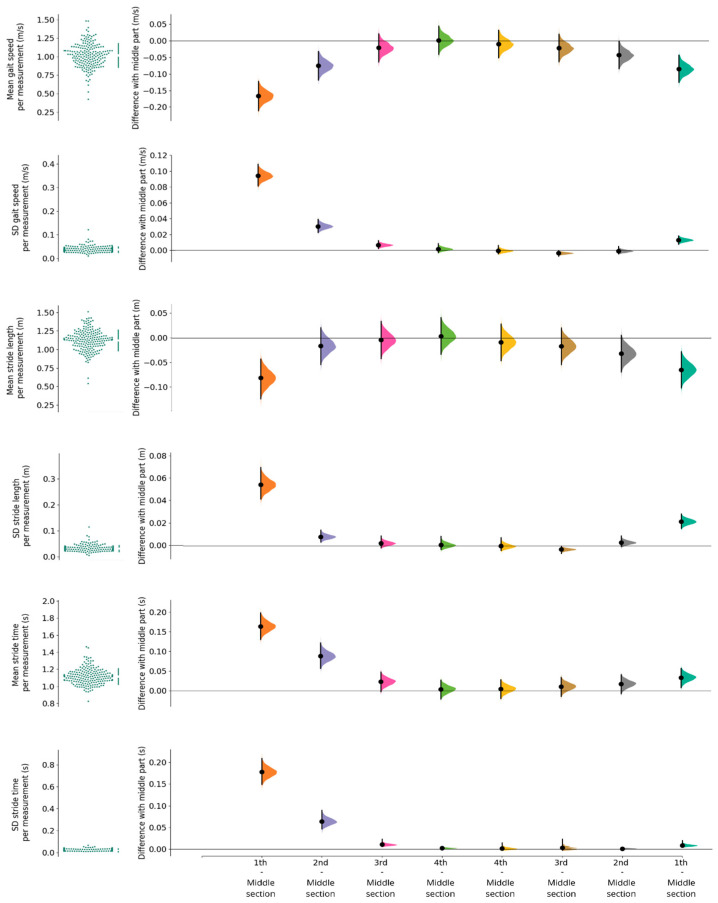
Subplots of the means and SDs of the strides in the middle section of the walking trajectory, and the mean differences and associated 99% CI of the first 4 strides around a turn. The first 4 strides after a turn show increasing speed and stride length with decreasing stride time. The last 4 strides before a turn show decreasing speed and stride length with increasing stride time. The difference between these strides with the mean gait speed, stride length and stride time of the middle section of the walkway are plotted in the lower subplots.

Figure 5 shows the average gait speed, stride length and stride time over all subjects of the first 4 strides after a turn and the last 4 strides before a turn. When comparing the 99% CIs, substantially decreased values for gait speed in the first two strides following the turn were found compared to the middle portion of walking. This was the result of higher stride times and—although to a lesser extent—lower stride length. The subsequent third stride showed some overlap with the middle part. In the strides before a turn, a similar, but reversed, trend was seen but with smaller mean differences, and overlap with steady-state gait was already visible for the second stride before the turn. Mean differences of SDs showed strikingly similar patterns. Table A2 of the Appendix A includes all means and SDs of gait speed, stride length and stride time for each of the four strides around a turn and the strides in the middle section of the walking trajectory, as well as their differences with the strides in the middle section of the walking trajectory. No differences in mean and SD of the gait speed between the three age groups and between the three speed groups were found (Appendix B).

## 4. Discussion

Unsurprisingly, our definition analysis showed that excluding strides based on different methods affected the means and variance of the spatiotemporal gait parameters. Excluding only the first and last stride around each turn (*ONE*), or through speed-based outlier analysis (*SPEED*), yielded highly similar means and variance of spatiotemporal gait parameters compared to the more conservative method, excluding two strides around each turn (*REFERENCE*). Including all strides in the straight-ahead portion of gait (*ALL*) or all strides that are at least 63% of the median stride length (*LENGTH*) seemed too lenient, including too much of the acceleration and deceleration phases. The in-depth analysis indicated that the first two strides after and last two strides before a turn were different from the steady-state walking period. Furthermore, strides after the turn (e.g., acceleration phase) had a more significant effect on the calculated spatiotemporal parameters compared to the strides before the turn (deceleration phase).

The absolute differences between the means of the spatiotemporal gait parameters of the five methods were very limited. The maximum mean deviation was 0.03 m/s in gait speed, 2 cm in stride length, and 20 ms in stride time (*ALL*). Nonetheless, the first stride after the turn was on average 0.17 m/s slower compared to the middle section, while the second stride before and the first stride after a turn were also considerably slower: 0.08 m/s. This suggests that these deviating strides had a limited effect on the mean, likely due to the relatively high number of strides in the middle section (n = 108 strides) compared to the number of first and second strides around turns (n = 10 turns). Importantly, it should be noted that the effect of including strides around the turn may be different when using other, mainly shorter, walking trajectories than our 10 m walkway.

In contrast to the means, marked differences between selection methods were observed for the variance of the gait parameters. To illustrate, including all strides in the analysis doubled the SD of gait speed from 0.04 m/s to 0.08 m/s. Although the *ONE* and *SPEED* methods resulted in almost similar SD values (for example ~0.01 m/s mean difference for gait speed), the 99% CI of the mean difference was still above 0, suggesting a consistent effect on the variance. Researchers and clinicians interested in the variability measures of gait should therefore proceed with extra caution in their decision making regarding the inclusion of strides for the analysis of gait.

The *SPEED* and *LENGTH* methods in our analysis could be seen as a form of outlier analysis. As definition *LENGTH* included almost all strides in the straight-ahead portion, *LENGTH* revealed similar results to *ALL* and seemed too lenient. The *SPEED* definition resulted in very similar outcomes as *ONE* and *REFERENCE*, but with a higher number of strides per trial included. A potential downside of these outlier analyses is that strides during the steady-state gait are excluded. This can be the case in patients with high variability in their gait pattern, for example, due to freezing of the gait in Parkinson’s disease. Due to the high variability, an inappropriate number of strides might get identified as outliers and as such get discarded by these methods, even though these strides might be highly interesting, and exclusion could be problematic. Additional data analysis on a group with a higher variability in their gait pattern is recommended to determine the effect of the different methods on the variance of spatiotemporal gait parameters.

Our in-depth analysis of the four strides before and after each turn showed differences in the first two strides around each turn compared to the middle section of the trajectory. Furthermore, the acceleration phase after a turn seems to affect the spatiotemporal gait parameters more than the deceleration phase before a turn. It should be noted that the ability to accelerate during gait initiation or decelerate to accommodate turning can be impacted by gait impairments due to age-related deficits. Muir et al. reported that adults over 80 years old needed more steps to reach their steady gait speed compared to the younger adults [10]. Besides age-related gait difficulties, a number of factors can impact the ability to accelerate during gait initiation, or deceleration before a turn, including pain or motor problems stemming from neurological or musculoskeletal diseases. Almost without exception, such impairments result in lower gait speed. To test if age-related or other factors impacting gait speed confounded our results, we compared subgroups regarding age and gait speed. This supplementary analysis did not provide evidence that age or factors affecting gait speed impacted the ability to accelerate or decelerate (Appendix B). Nonetheless, as our sample size was restricted to individuals with OA, we cannot rule out that these findings do not translate to people with more severe gait impairments, such as in people after stroke or with Parkinson’s disease.

As mentioned above, the influence of excluding more or less strides around turns is also dependent on how many strides are collected as part of the steady-state gait (i.e., the included strides). This number depends on the length of the walkway, the total testing time, and gait speed of an individual. To illustrate, excluding two strides at both ends (*REFERENCE*) of a ten-meter walkway will leave approximately six meters for steady-state gait. As the stride length in this study was 1.12 m on average, this would result in the inclusion of five strides per leg on average per stretch of the trajectory. In settings where shorter walkways are used, assessors could estimate in advance how many stretches would be needed to obtain the number of strides necessary for their specific research or clinical purpose.

This study includes some limitations that merit attention. First, the study population was restricted to people with OA in the lower extremities. Even though a large data set of 199 measurements was used, caution should be exercised when translating these results to other groups with gait impairments, as also laid out above. Secondly, various components of the algorithm for gait analysis were validated in previous studies [7,9,14,15], but a validity study for the entire algorithm for this set-up is still in progress (with promising results). Thirdly, only basic spatiotemporal gait parameters were analyzed. The effect of stride selection on other kinematic or non-linear dynamic measures warrants investigation in future studies.

In conclusion, our analyses suggest that the first two strides during the acceleration and last two strides during the deceleration phases around turns should not be included. Nevertheless, the specific aims of the gait assessment and available test conditions should guide the decision on which selection method to use to select strides representative of the preferred, steady-state gait.

## Figures and Tables

**Table 1:** Participant characteristics.

Participant Characteristics	
N total ^1^	191
Male/female (N)	81/110
Age (mean ± SD years)	63.1 ± 9.0
End-stage OA/post surgery (N) ^1^	93/106
Hip/Knee/Ankle OA (N)	71/117/3
Height (mean ± SD cm)	173.8 ± 9.7
Weight (mean ± SD kg)	85.4 ± 15.8

^1^ Eight participants had two measurements (pre- and post-surgery). OA: osteoarthritis.

**Figure A1:**
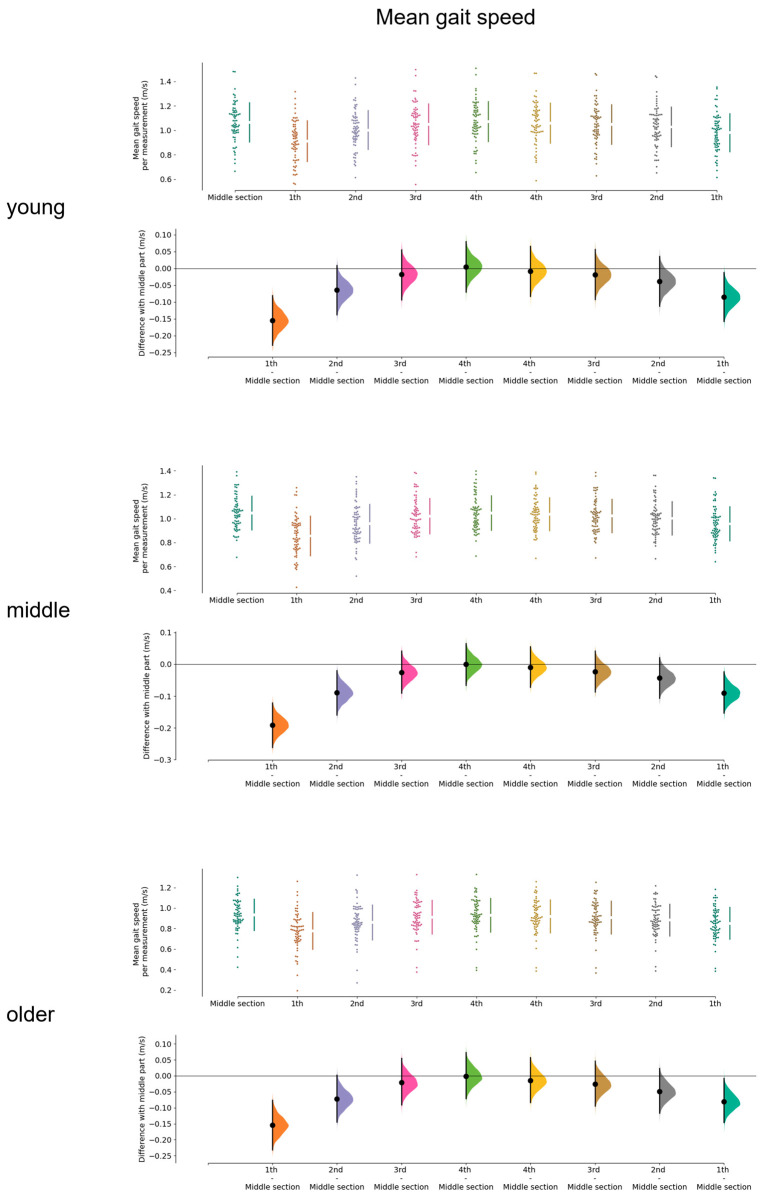
Mean gait speed of the different participants during the walking trajectory, and the mean differences and associated 99% CI of the first and last 4 strides around a turn. The top subplot includes data of the young age group, the middle subplot, the middle age group, and the bottom subplot, the older age group.

**Figure A2:**
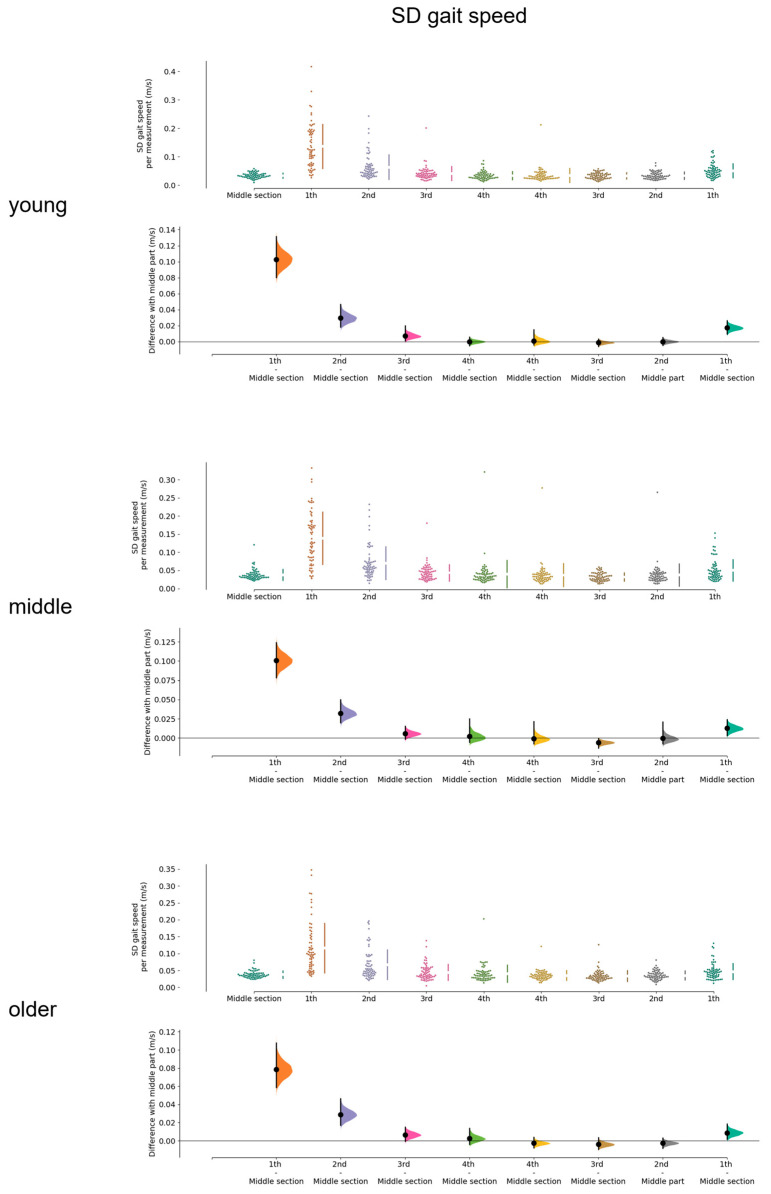
SD of the different participants during the walking trajectory, and the mean differences and associated 99% CI of the first and last 4 strides around a turn. The top subplot includes data of the young age group, the middle subplot, the middle age group, and the bottom subplot, the older age group.

**Figure A3:**
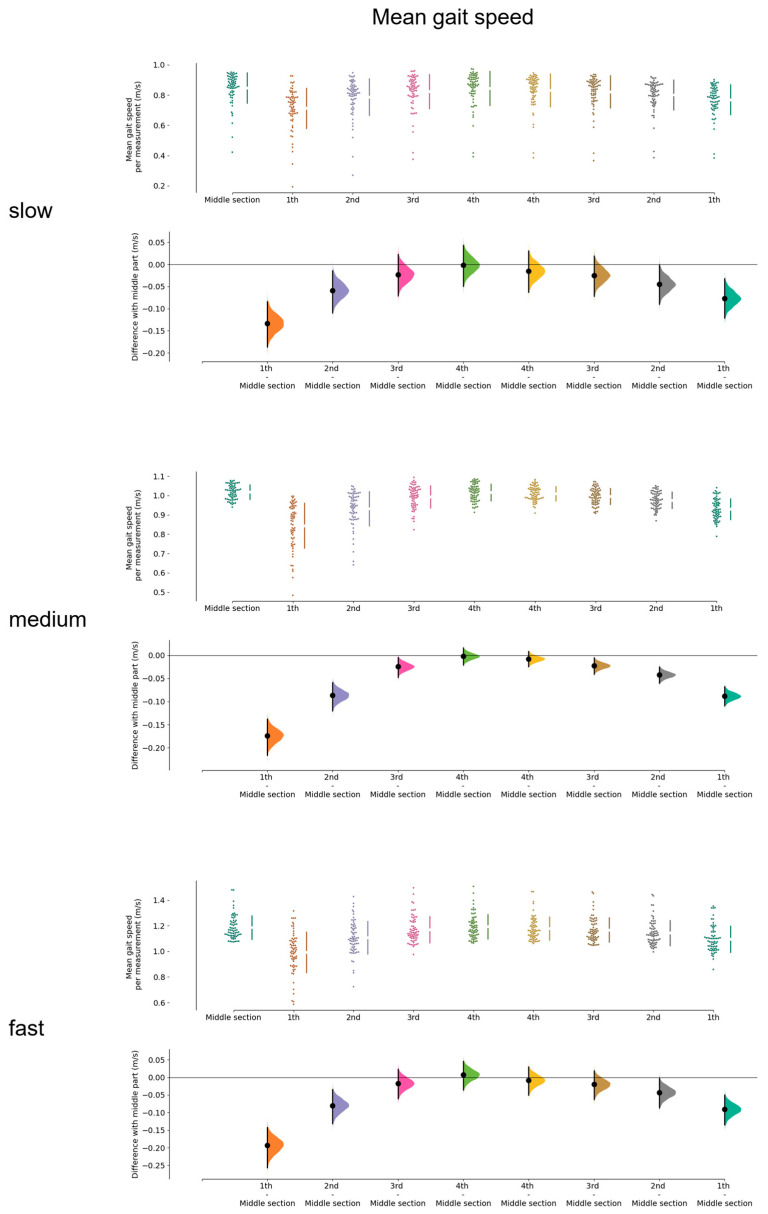
Mean gait speed of the different participants during the walking trajectory, and the mean differences and associated 99% CI of the first and last 4 strides around a turn. The top subplot includes data of the slow gait speed group, the middle subplot of the medium gait speed group and the bottom subplot of the fast gait speed group.

**Figure A4:**
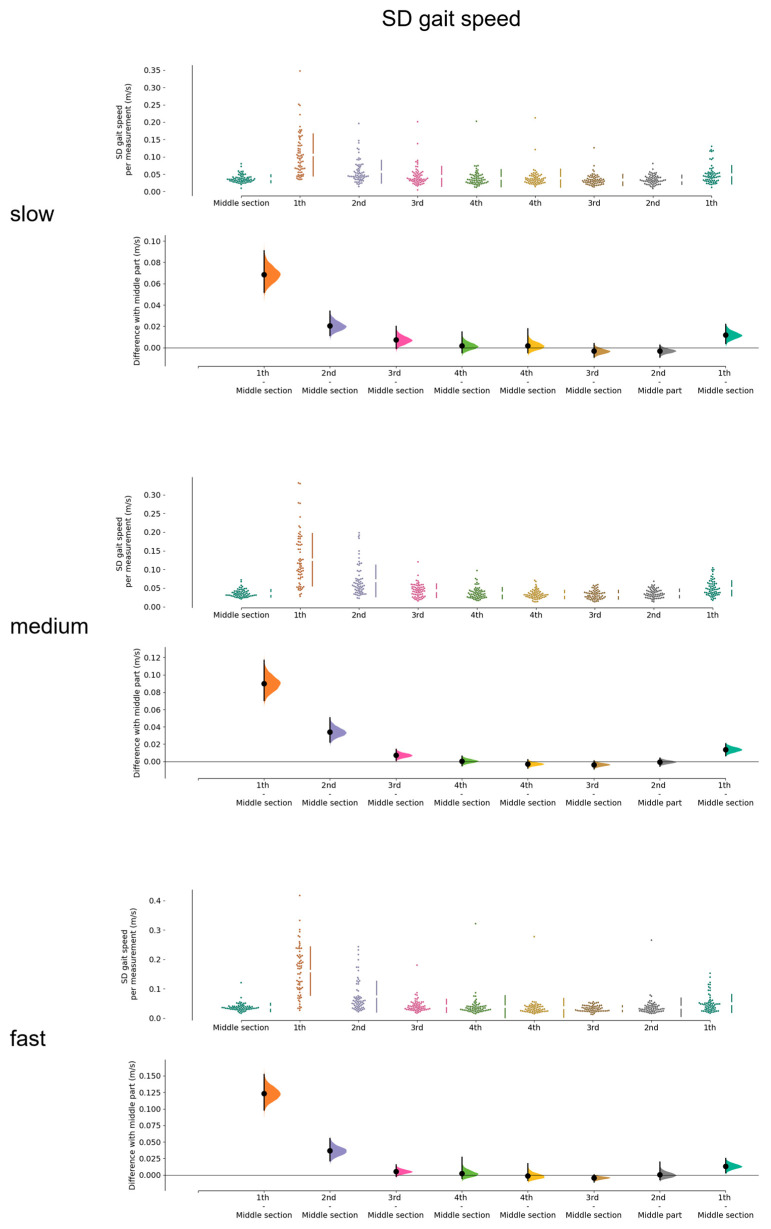
SD of the different participants during the walking trajectory, and the mean differences and associated 99% CI of the first and last 4 strides around a turn. The top subplot includes data of the slow gait speed group, the middle subplot of the medium gait speed group and the bottom subplot of the fast gait speed group.

## Data Availability

Data are available upon request.

## Appendix A

**Table A1:** Mean [99% CI] across subjects and the average within subject SD [99% CI] for gait speed, stride length, stride time and number of strides for each definition.

	Mean REFERENCE	Mean ALL	Mean Difference ALL− REFERENCE	Mean ONE	Mean Difference ONE− REFERENCE	Mean LENGTH	Mean Difference LENGTH− REFERENCE	Mean SPEED	Mean Difference SPEED− REFERENCE
Mean gait speed (m/s)	1.01 [0.98; 1.04]	0.99 [0.96; 1.02]	−0.03 [−0.07; 0.02]	1.00 [0.97; 1.03]	−0.01 [−0.05; 0.03]	0.99 [0.96; 1.02]	−0.02 [−0.06; 0.02]	1.01 [0.98; 1.04]	−0.00 [−0.05; 0.04]
SD gait speed (m/s)	0.04 [0.04; 0.04]	0.08 [0.08; 0.09]	0.04 [0.04; 0.05]	0.05 [0.05; 0.06]	0.01 [0.01; 0.02]	0.07 [0.07; 0.08]	0.03 [0.03; 0.04]	0.04 [0.04; 0.04]	−0.00 [−0.01; −0.00]
Mean stride length (m)	1.12 [1.1; 1.15]	1.11 [1.08; 1.13]	−0.02 [−0.05; 0.02]	1.12 [1.09; 1.14]	−0.00 [−0.04; 0.03]	1.11 [1.08; 1.14]	−0.01 [−0.05; 0.02]	1.12 [1.09; 1.15]	−0.00 [−0.04; 0.03]
SD stride length (m)	0.04 [0.03; 0.04]	0.07 [0.06; 0.07]	0.03 [0.03; 0.04]	0.04 [0.04; 0.04]	0.00 [−0.00; 0.01]	0.05 [0.05; 0.06]	0.02 [0.01; 0.02]	0.04 [0.03; 0.04]	0.00 [−0.00; 0.00]
Mean stride time (s)	1.12 [1.1; 1.13]	1.14 [1.12; 1.16]	0.02 [−0.00; 0.04]	1.13 [1.11; 1.14]	0.01 [−0.02; 0.03]	1.14 [1.12; 1.15]	0.02 [−0.00; 0.04]	1.12 [1.1; 1.14]	0.00 [−0.02; 0.03]
SD stride time (s)	0.03 [0.02; 0.03]	0.09 [0.08; 0.1]	0.06 [0.05; 0.07]	0.05 [0.04; 0.05]	0.02 [0.01; 0.03]	0.08 [0.07; 0.09]	0.06 [0.05; 0.07]	0.03 [0.03; 0.03]	0.00 [−0.00; 0.00]
Mean strides (N)	108 [105; 112]	160 [157; 164]	51 [48; 57]	134 [130; 137]	25 [21; 31]	159 [156; 163]	51 [47; 56]	135 [132; 138]	27 [22; 32]

**Table A2:** Mean [99% CI] of gait speed, stride length, and stride time of the first four strides after and before a turn, and the middle section of the walking trajectory. Included are the difference (diff.) of these strides with the middle section.

	Middle Section	Strides after Turn								Stride Before Turn							
	Mean	Mean 1th	Mean Diff. 1th— Middle Section	Mean 2nd	Mean Diff. 2nd— Middle Section	Mean 3rd	Mean Diff. 3rd— Middle Section	Mean 4th	Mean Diff. 4th— Middle Section	Mean 4th	Mean Diff. 4th— Middle Section	Mean 3rd	Mean Diff. 3rd— Middle Section	Mean 2nd	Mean Diff. 2nd— Middle Section	Mean 1th	Mean Diff. 1th— Middle Section
Mean gait speed (m/s)	1.02 [0.99; 1.05]	0.85 [0.82; 0.88]	−0.17 [−0.21; −0.12]	0.94 [0.91; 0.97]	−0.08 [−0.12; −0.03]	0.99 [0.96; 1.03]	−0.02 [−0.06; 0.02]	1.02 [0.99; 1.05]	0.00 [−0.04; 0.04]	1.01 [0.98; 1.04]	−0.01 [−0.05; 0.03]	0.99 [0.96; 1.02]	−0.02 [−0.06; 0.02]	0.97 [0.94; 1.0]	−0.04 [−0.08; −0.00]	0.93 [0.9; 0.96]	−0.09 [−0.13; −0.04]
SD gait speed (m/s)	0.04 [0.03; 0.04]	0.13 [0.12; 0.14]	0.09 [0.08; 0.11]	0.07 [0.06; 0.08]	0.03 [0.02; 0.04]	0.04 [0.04; 0.05]	0.01 [0.00; 0.01]	0.04 [0.03; 0.04]	0.00 [−0.00; 0.01]	0.04 [0.03; 0.04]	−0.00 [−0.00; 0.01]	0.03 [0.03; 0.04]	−0.00 [−0.01; −0.00]	0.04 [0.03; 0.04]	−0.00 [−0.00; 0.00]	0.05 [0.04; 0.05]	0.01 [0.01; 0.02]
Mean stride length (m)	1.12 [1.10; 1.15]	1.04 [1.01; 1.08]	−0.08 [−0.12; −0.04]	1.11 [1.08; 1.14]	−0.02 [−0.05; 0.02]	1.12 [1.09; 1.15]	−0.00 [−0.04; 0.03]	1.13 [1.1; 1.16]	0.00 [−0.03; 0.04]	1.12 [1.09; 1.14]	−0.02 [−0.05; 0.03]	1.11 [1.08; 1.13]	−0.02 [−0.05; 0.02]	1.09 [1.07; 1.12]	−0.03 [−0.07; 0.01]	1.06 [1.03; 1.09]	−0.06 [−0.10; −0.03]
SD stride length (m)	0.03 [0.03; 0.03]	0.09 [0.07; 0.1]	0.05 [0.04; 0.07]	0.04 [0.04; 0.04]	0.01 [0.00; 0.01]	0.03 [0.03; 0.04]	0.00 [−0.00; 0.01]	0.03 [0.03; 0.04]	0.00 [−0.00; 0.01]	0.03 [0.03; 0.04]	−0.00 [−0.00; 0.01]	0.03 [0.03; 0.03]	−0.00 [−0.01; −0.00]	0.03 [0.03; 0.04]	0.00 [−0.00; 0.01]	0.05 [0.05; 0.06]	0.02 [0.02; 0.03]
Mean stride time (s)	1.12 [1.1; 1.13]	1.28 [1.25; 1.31]	0.16 [0.13; 0.20]	1.20 [1.18; 1.23]	0.09 [0.06; 0.12]	1.14 [1.12; 1.16]	0.02 [−0.00; 0.04]	1.12 [1.1; 1.14]	0.00 [−0.02; 0.03]	1.12 [1.1; 1.14]	0.00 [−0.02; 0.03]	1.13 [1.11; 1.14]	0.01 [−0.01; 0.03]	1.13 [1.12; 1.15]	0.02 [−0.01; 0.04]	1.15 [1.13; 1.17]	0.03 [0.01; 0.06]
SD stride time (s)	0.02 [0.02; 0.02]	0.20 [0.17; 0.23]	0.18 [0.15; 0.21]	0.09 [0.06; 0.11]	0.06 [0.05; 0.09]	0.03 [0.03; 0.04]	0.01 [0.01; 0.02]	0.02 [0.02; 0.03]	0.00 [−0.00; 0.01]	0.02 [0.02; 0.03]	0.00 [−0.00; 0.01]	0.02 [0.01; 0.03]	0.00 [−0.00; 0.02]	0.02 [0.02; 0.03]	0.00 [−0.00; 0.00]	0.03 [0.02; 0.04]	0.01 [0.00; 0.02]

## Appendix B. Analysis for Impact of Age and Gait Ability

### Appendix B.1. Methods

To explore if stride-selection definitions should be different depending on age or difficulty, we analyzed the acceleration and deceleration phases for different age and gait speed groups. We split the research sample into tertiles based on age and on gait speed, as achieved in the middle section of the walking trajectory. The first four strides after a turn and last four strides before a turn were compared to the middle section of the walking trajectory using mean differences and their 99% CI. The middle section of the walking trajectory consisted of the fifth stride after the turn, up to and including the fifth stride before the next turn. In each subgroup, we performed shared control mean difference statistical tests (ordered groups ANOVA) of Python’s dabest (v0.3.1) package [19].

### Appendix B.2. Results

The results of the mean and SD of gait speed for the different age groups for the first strides after a turn, and the last strides before turning are presented in Figure A1 and Figure A2. Results of the mean and SD of gait speed for the different speed groups are presented in Figure A3 and Figure A4. The mean differences between the first strides after and last stride before a turn and the middle section were very similar between age groups and speed groups. This was true for the mean of gait speed as well as the SD of gait speed. Similar results were found for the stride time and stride length.
